# Supplementary material for: OpenMM 7: Rapid development of high performance algorithms for molecular dynamics
Source: PLoS Comput Biol. 2017 Jul 26;13(7):e1005659. doi: 10.1371/journal.pcbi.1005659 (PMC5549999; doi:10.1371/journal.pcbi.1005659)
Supplement: S1 Source Code — (ZIP) [file pcbi.1005659.s002.zip › OpenMM-7.0.1-Source/docs/api-c++/generated/OpenMM.AmoebaOutOfPlaneBendForce.html]

AmoebaOutOfPlaneBendForce — OpenMM @OPENMM\_MAJOR\_VERSION@.@OPENMM\_MINOR\_VERSION@ documentation


# AmoebaOutOfPlaneBendForce¶

*class* `OpenMM::``AmoebaOutOfPlaneBendForce`¶
:   This class implements the Amoeba out-of-plane bend interaction.

    To use it, create an OutOfPlaneBendForce object then call `addOutOfPlaneBend()` once for each outOfPlaneBend. After an out-of-plane bend has been added, you can modify its force field parameters by calling `setOutOfPlaneBendParameters()`. This will have no effect on Contexts that already exist unless you call `updateParametersInContext()`.

    Methods

    |  |  |
    | --- | --- |
    | `AmoebaOutOfPlaneBendForce` | Create an `AmoebaOutOfPlaneBendForce`. |
    | `getNumOutOfPlaneBends` | Get the number of out-of-plane bend terms in the potential function |
    | `setAmoebaGlobalOutOfPlaneBendCubic` | Set the global cubic term |
    | `getAmoebaGlobalOutOfPlaneBendCubic` | Get the global cubic term |
    | `setAmoebaGlobalOutOfPlaneBendQuartic` | Set the global cubic term |
    | `getAmoebaGlobalOutOfPlaneBendQuartic` | Get the global quartic term |
    | `setAmoebaGlobalOutOfPlaneBendPentic` | Set the global pentic term |
    | `getAmoebaGlobalOutOfPlaneBendPentic` | Get the global pentic term |
    | `setAmoebaGlobalOutOfPlaneBendSextic` | Set the global sextic term |
    | `getAmoebaGlobalOutOfPlaneBendSextic` | Get the global sextic term |
    | `addOutOfPlaneBend` | Add an out-of-plane bend term to the force field. |
    | `getOutOfPlaneBendParameters` | Get the force field parameters for an out-of-plane bend term. |
    | `setOutOfPlaneBendParameters` | Set the force field parameters for an out-of-plane bend term. |
    | `updateParametersInContext` | Update the per-bend term parameters in a `Context` to match those stored in this `Force` object. |
    | `usesPeriodicBoundaryConditions` | Returns whether or not this force makes use of periodic boundary conditions. |

    `AmoebaOutOfPlaneBendForce`()¶
    :   Create an `AmoebaOutOfPlaneBendForce`.

    int `getNumOutOfPlaneBends`() *const*¶
    :   Get the number of out-of-plane bend terms in the potential function

    void `setAmoebaGlobalOutOfPlaneBendCubic`(double *cubicK*)¶
    :   Set the global cubic term

        |  |  |
        | --- | --- |
        | Parameters: |  |

        - **cubicK** – the cubic force constant for the angle

    double `getAmoebaGlobalOutOfPlaneBendCubic`() *const*¶
    :   Get the global cubic term

        |  |  |
        | --- | --- |
        | Returns: | global cubicK term |

    void `setAmoebaGlobalOutOfPlaneBendQuartic`(double *quarticK*)¶
    :   Set the global cubic term

        |  |  |
        | --- | --- |
        | Parameters: |  |

        - **quarticK** – the quartic force constant for the angle

    double `getAmoebaGlobalOutOfPlaneBendQuartic`() *const*¶
    :   Get the global quartic term

        |  |  |
        | --- | --- |
        | Returns: | global quartic term |

    void `setAmoebaGlobalOutOfPlaneBendPentic`(double *penticK*)¶
    :   Set the global pentic term

        |  |  |
        | --- | --- |
        | Parameters: |  |

        - **penticK** – the pentic force constant for the angle

    double `getAmoebaGlobalOutOfPlaneBendPentic`() *const*¶
    :   Get the global pentic term

        |  |  |
        | --- | --- |
        | Returns: | global penticK term |

    void `setAmoebaGlobalOutOfPlaneBendSextic`(double *sexticK*)¶
    :   Set the global sextic term

        |  |  |
        | --- | --- |
        | Parameters: |  |

        - **sexticK** – the sextic force constant for the angle

    double `getAmoebaGlobalOutOfPlaneBendSextic`() *const*¶
    :   Get the global sextic term

        |  |  |
        | --- | --- |
        | Returns: | global sexticK term |

    int `addOutOfPlaneBend`(int *particle1*, int *particle2*, int *particle3*, int *particle4*, double *k*)¶
    :   Add an out-of-plane bend term to the force field.

        |  |  |
        | --- | --- |
        | Parameters: |  |

        - **particle1** – the index of the first particle connected by the outOfPlaneBend
        - **particle2** – the index of the second particle connected by the outOfPlaneBend
        - **particle3** – the index of the third particle connected by the outOfPlaneBend
        - **particle4** – the index of the fourth particle connected by the outOfPlaneBend
        - **k** – the force constant for the out-of-plane bend

        |  |  |
        | --- | --- |
        | Returns: | the index of the out-of-plane bend that was added |

    void `getOutOfPlaneBendParameters`(int *index*, int &*particle1*, int &*particle2*, int &*particle3*, int &*particle4*, double &*k*) *const*¶
    :   Get the force field parameters for an out-of-plane bend term.

        |  |  |
        | --- | --- |
        | Parameters: |  |

        - **index** – the index of the outOfPlaneBend for which to get parameters
        - **particle1** – [out] the index of the first particle connected by the outOfPlaneBend
        - **particle2** – [out] the index of the second particle connected by the outOfPlaneBend
        - **particle3** – [out] the index of the third particle connected by the outOfPlaneBend
        - **particle4** – [out] the index of the fourth particle connected by the outOfPlaneBend
        - **k** – [out] the force constant for the out-of-plane bend

    void `setOutOfPlaneBendParameters`(int *index*, int *particle1*, int *particle2*, int *particle3*, int *particle4*, double *k*)¶
    :   Set the force field parameters for an out-of-plane bend term.

        |  |  |
        | --- | --- |
        | Parameters: |  |

        - **index** – the index of the outOfPlaneBend for which to set parameters
        - **particle1** – the index of the first particle connected by the outOfPlaneBend
        - **particle2** – the index of the second particle connected by the outOfPlaneBend
        - **particle3** – the index of the third particle connected by the outOfPlaneBend
        - **particle4** – the index of the fourth particle connected by the outOfPlaneBend
        - **k** – the force constant for the out-of-plane bend

    void `updateParametersInContext`(Context &*context*)¶
    :   Update the per-bend term parameters in a `Context` to match those stored in this `Force` object. This method provides an efficient method to update certain parameters in an existing `Context` without needing to reinitialize it. Simply call `setOutOfPlaneBendParameters()` to modify this object’s parameters, then call `updateParametersInContext()` to copy them over to the `Context`.

        The only information this method updates is the values of per-bend term parameters. The set of particles involved in a term cannot be changed, nor can new terms be added.

    bool `usesPeriodicBoundaryConditions`() *const*¶
    :   Returns whether or not this force makes use of periodic boundary conditions.

        |  |  |
        | --- | --- |
        | Returns: | true if nonbondedMethod uses PBC and false otherwise |

High performance molecular simulation on GPUs


### Navigation

- Core Objects
- Forces
- Integrators
- Extras

©2015, Stanford University and the Authors.
|
Powered by Sphinx 1.3.1
& Alabaster 0.7.7
|
Page source
